# Uncoupling fate: Klotho—Goddess of fate and regulator of life and ageing

**DOI:** 10.1111/ajag.12772

**Published:** 2020-07-20

**Authors:** Michael Lichtenauer, Anne‐Katrin Altwein, Kristen Kopp, Hermann Salmhofer

**Affiliations:** ^1^ Division of Cardiology Department of Internal Medicine II Paracelsus Medical University Salzburg Austria; ^2^ Atelier Eichenallee Ivenack Germany; ^3^ Divisions of Nephrology and Endocrinology Department of Internal Medicine I Paracelsus Medical University Salzburg Austria

The Greek goddess Klotho (or Clotho) represents fate in ancient mythology. She has been celebrated in art over the centuries and today has found a role as the Klotho protein in biomedical research. Will the 21st century see an uncoupling of human destiny from the fates of old?

## 1 KLOTHO IN GREEK MYTHOLOGY

Klotho was one of the three Fates in Greek mythology. Klotho and her two sisters, Lachesis and Atropos, controlled the thread of life of each human being.

Klotho was responsible for spinning the thread of life. She also possessed the power to choose who was saved or put to death. It was Klotho who brought Pelops back to life after his father had killed him, cut him into pieces, boiled him and had him served to the gods. Zeus ordered Klotho to ritually restore Pelops’ body and bring him back to life. In another story, about King Admetus and his wife Alcestis, Apollo persuaded the Fates to grant Admetus a reprieve from his fated day of death (in fact Apollo made the Fates drunk) if he could find someone to die instead of him.

Lachesis, the second of the three Fates, was responsible for measuring the length of the thread and deciding how much time of life was allowed for each human being.

The third Fate Atropos was the oldest of the three sisters. She was the one who chose the mechanism of death and how the life of a mortal ended by cutting the thread of life with her shears.

It was said that the three Fates appear within three days of someone's birth to decide their fate. Klotho's place in Greek mythology alongside her sisters was significant.

## 2 KLOTHO IN THE VISUAL ARTS

The first representation of Klotho in the arts dates back to ancient Greece. Klotho, Lachesis and Atropos were often depicted as old women who were bitter and showed no mercy. Usually, they are pictured with their known attributes, Klotho with the thread of life on a spindle, Lachesis with a tape measure or eye glass and Atropos with a pair of scissors.

**Figure 1:**
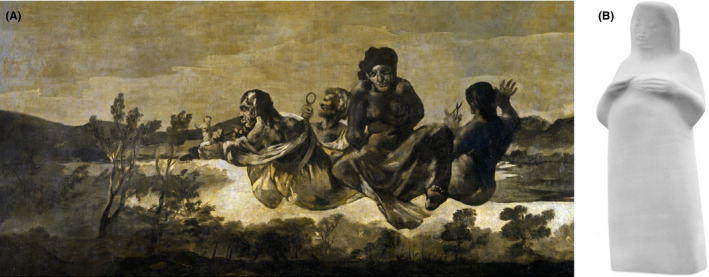
(A) Atropos/The Fates. Francisco de Goya (1819‐1823), oil mural transferred to canvas (123cm × 266cm), Museo del Prado, Madrid. (B) Clotho, Three Goddesses of Fate: Clotho who spins the thread of life. Anne‐Katrin Altwein, marble, height 250 cm, Paradies Park, City of Jena

The three Fates also feature in one of Francisco de Goya's famous black paintings. During his late period, Goya painted fourteen intense and haunting paintings on the walls of his house (the Quinta del Sordo, House of the Deaf Man) where he lived in near‐total isolation, disillusioned by the political developments in Spain at that time. The painting shows three female figures floating in the air thought to be the three Fates. Klotho is not shown with a spindle but with a doll or maybe a newborn child. Lachesis is depicted looking through a lens measuring the length of the thread of life. Atropos is shown carrying a pair of scissors ready to cut the thread of life. A fourth figure is pictured with his hands bound behind him as if being captive. It is speculated that this figure might represent Prometheus or an unknown man waiting for the three Fates to decide his destiny (Figure 1A).

In recent times, Klotho, Lachesis and Atropos are portrayed by German artist Anne‐Katrin Altwein in three sculptures (sculpture Klotho, Figure 1B). The sculptures were initially erected at the entrance to the University Hospital Jena in Thuringia, Germany. Their current location is the Paradies Park in the centre of the city of Jena. The artist has chosen the subject of the three Fates from Greek mythology because she felt the campus of the University Hospital Jena should provide creative inspiration for patients strolling in the recreation area of the hospital. In the sculpture, Klotho is not shown with her usual attributes, but as a pregnant woman, as the artist sought to portray the goddess in a more positive light, perhaps subverting her reputation as fate.

All of us remain keen to know our own destiny, so it is not surprising that the three Fates have been a subject for artists from around the world from ancient times until today.

## 3 THE ROLE OF KLOTHO PROTEIN IN AGEING

Klotho, in the form of a protein, has been introduced to the world of molecular biology. The Klotho protein is a β‐glucuronidase discovered in 1997 by Kuro‐o et al at the National Institute of Neuroscience, Tokyo.^1^ Kuro‐o et al have also found that Klotho is an ageing‐suppressor gene. Genetic defects in the Klotho gene lead to a specific syndrome in mice that resembles premature ageing, with a shortening in lifespan, ectopic calcification and increased arteriosclerosis. Over‐expression of Klotho has been shown to inhibit the ageing process in mice resulting in an increased life span by 20%‐30%. The gene and its associated protein were named Klotho because of their role in determining life span.

The Klotho gene encodes for a transmembrane protein and is expressed in multiple tissues, primarily the kidney. Further research has shown that Klotho functions as a co‐receptor for the binding of fibroblast growth factor 23 (FGF‐23) to the FGF receptors (FGFR) by forming a Klotho/FGFR/FGF‐23 complex, leading to activation of FGF‐23 signalling. It has been shown that circulatory Klotho levels are significantly lower in patients with chronic kidney disease (CKD) whereas FGF‐23 is elevated.

In older humans, higher circulating Klotho concentrations relate to longevity, better physical performance, lower disability, morbidity and cognitive decline. Shardell found that higher plasma Klotho concentrations were associated with lower likelihoods of frailty and particularly exhaustion.^2^ Genetic research suggests Klotho may be able to be upregulated.

Klotho protein, FGF‐23 (Lachesis) and vitamin D (Atropos) have been styled as the three sisters of fate by Ellidag et al, investigating these three factors in patients with multiple sclerosis. Klotho might prove to be a novel therapeutic strategy for a broad spectrum of life‐threatening diseases, either as a protein supplement or more likely by genetic upregulation. Klotho might also be a useful biomarker for diagnosis and risk stratification in patients with frailty, renal impairment and cardiovascular disease.

Klotho's role in our destiny was undisputed by the ancient Greeks. Depictions of the three Fates in art and sculpture over the centuries have kept the myth of Klotho alive. Recent scientific advances in the molecular biology of ageing in which a gene and its associated protein have been named after Klotho perpetuate the importance of the concept of destiny and our desire to transcend our fate. Perhaps with further research in the twenty‐first century, we will be able to uncouple human destiny from the Fates of old.

## CONFLICT OF INTEREST

No conflicts of interest declared.
